# Mite Allergen Der-p2 Triggers Human B Lymphocyte Activation and Toll-Like Receptor-4 Induction

**DOI:** 10.1371/journal.pone.0023249

**Published:** 2011-09-06

**Authors:** Jaw Ji Tsai, Shing Hwa Liu, Sui Chu Yin, Cheng Ning Yang, Hong Sheng Hsu, Wen Bao Chen, En Chih Liao, Wen Jane Lee, Hung Chuan Pan, Meei Ling Sheu

**Affiliations:** 1 Institute of Biomedical Sciences, National Chung Hsing University, Taichung, Taiwan; 2 Department of Education and Research, Taichung Veterans General Hospital, Taichung, Taiwan; 3 Institute of Toxicology, College of Medicine, National Taiwan University, Taipei, Taiwan; 4 Institute of Neuroscience, School of Life Science, National Yang-Ming University, Taipei, Taiwan; University of Pennsylvania School of Medicine, United States of America

## Abstract

**Background:**

Allergic disease can be characterized as manifestations of an exaggerated inflammatory response to environmental allergens triggers. Mite allergen Der-p2 is one of the major allergens of the house dust mite, which contributes to TLR4 expression and function in B cells in allergic patients. However, the precise mechanisms of Der-p2 on B cells remain obscure.

**Methodology/Principal Findings:**

We investigated the effects of Der-p2 on proinflammatory cytokines responses and Toll-like receptor-4 (TLR4)-related signaling in human B cells activation. We demonstrated that Der-p2 activates pro-inflammatory cytokines, TLR4 and its co-receptor MD2. ERK inhibitor PD98059 significantly enhanced TLR4/MD2 expression in Der-p2-treated B cells. Der-p2 markedly activated mitogen-activated protein kinase (MAPK) phosphatase-1 (MKP-1) and decreased p38 phosphorylation in B cells. MKP-1-siRNA downregulated TLR4/MD2 expression in Der-p2-treated B cells. In addition, Der-p2 significantly up-regulated expression of co-stimulatory molecules and increased B cell proliferation. Neutralizing Der-p2 antibody could effectively abrogate the Der-p2-induced B cell proliferation. Der-p2 could also markedly induce NF-κB activation in B cells, which could be counteracted by dexamethasone.

**Conclusions/Significance:**

These results strongly suggest that Der-p2 is capable of triggering B cell activation and MKP-1-activated p38/MAPK dephosphorylation-regulated TLR4 induction, which subsequently enhances host immune, defense responses and development of effective allergic disease therapeutics in B cells.

## Introduction

The house dust mite is a major source of environmental inhalation allergens involved in the pathogenesis of anaphylactic type reactions in humans and animals [1], [2]. The mite allergen *Dermatophagoides pteronyssinus* group-2 (Der-p2) is a major allergen and is highly correlated with asthma, atopic dermatitis and allergic rhinitis. It has been estimated that 79.2% of patients with asthma, wheezing and/or rhinitis have IgE antibodies to Der-p2 [3]. Recently, the Der-p2 allergen was found to show structural homology with MD-2 suggesting that Der-p2 tends to be targeted by adaptive immune responses because of its autoadjuvant properties [4]. The structure of Der-p2 has been suggested to provide a useful tool in the design of recombinant immunotherapeutics for the group-2 allergens [5]. Mycobacterium bovis-bacillus Calmette-Guerin (BCG) inoculation with Der-p2 has been shown to cause a Th1-type immune response that hinders Der-p2-induced allergic sensitization as well as the development of airway inflammation [6]. Moreover, human T cells conditioned by the proteolytic activity of mite allergen Der-p1 become empowered to trigger enhanced IgE synthesis by B cells [7]. B cells are known to exert a number of antibody-independent functions, capturing and concentrating antigen, producing cytokines, influencing responses of T cells and dendritic cells, contributing distinct functions during the immune response, affecting lymphoid tissue structures, and participating in tissue repair [8]–[10]. However, the precise action and mechanism underlying the effects of Der-p2 on B cell activation and immune response remain unclear.

 Both innate and adaptive mechanisms are commonly used by the host to detect and eliminate pathogenic microbes. In addition to its intrinsic capacities to mediate Toll-like receptors (TLR) expression and microbial destruction, B cells also establish an important link between the innate and adaptive branches of the immune system [11]–[14]. Activation of B cells by bacterial lipopolysaccharide (LPS) and peptidoglycan innate stimulation could induce TLR4 up-regulation. Functional deficiency or genetic deletion of TLR4 increased the susceptibility to *Haemophilus influenzae*, *Streptococcus pneumoniae*, and *Klebsiella pneumoniae*-related respiratory tract infections in murine models [15], [16]. TLR4 has also been demonstrated to play a crucial role in protection from acute lethal infection by *Leptospira interrogans serovar Icterohaemorrhagiae* and in the control of *leptospiral* burden during sublethal chronic infection [17]. However, Okumura and colleagues have shown that host TLR4 is a sensor for Ebola virus glycoprotein and that resultant TLR4-related signalings lead to the production of proinflammatory cytokines and suppressor of cytokine signaling 1 (SOCS1), which play a role in the immunopathogenesis of Ebola virus infection [18], [19]. These findings suggest that the activation of TLR4 seems to be required for protective immunity against infections, but also mediates the effects of systemic endotoxin infections. However, the effects of Der-p2 on TLR4 induction and TLR4-regulated immune responses in B cells remain unclear.

Mitogen-activated protein (MAP) kinases have been shown to be involved in all aspects of immune responses in mammalian species, from the initiation phase of innate immunity, to activation of adaptive immunity, and to cell death when immune function is complete [20]. MAP kinase phosphatase (MKP)-1, which is essential for the dephosphorylation/deactivation of MAP kinases p38 and JNK, has been demonstrated to be an essential feedback regulator of the innate immune response [21]–[24]. Nevertheless, the roles of MAP kinases and MKP-1 in Der-p2-regulated B cell activation and immune responses are still unknown. In view of the importance of B cell activation in regulating immune repertoires and responses, in this study, we investigated the precise actions and mechanisms of Der-p2 on B cell activation and TLR4 induction. The present study showed that Der-p2 specifically up-regulated MKP-1 expression and activity in human B cells, which in turn, resulted in p38/MAPK dephosphorylation and triggered TLR4 induction.

## Results

### Der-p2 activates proliferation and the expressions of proinflammatory cytokines and TLR4 in human B cells

**Figure 1:**
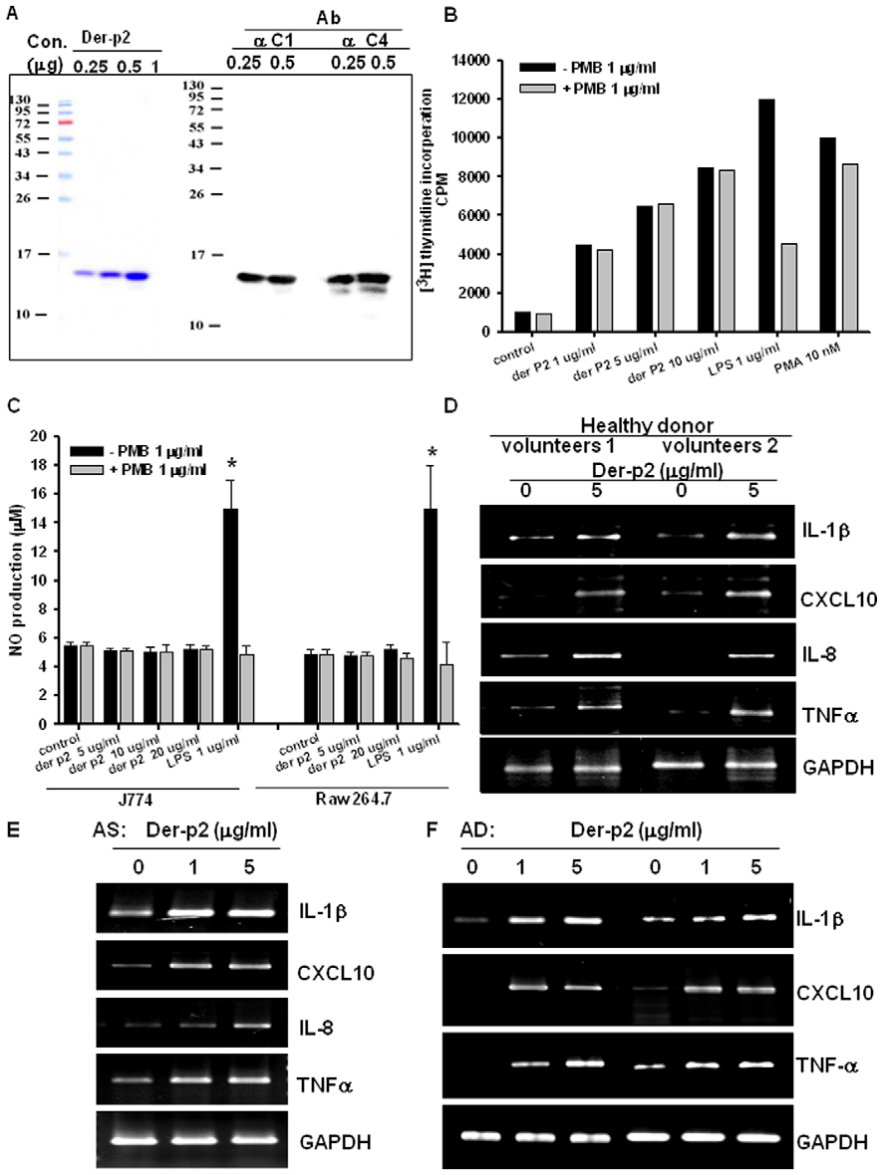
Mite allergen Der-p2 induces proliferation and expression of proinflammatory mediators in B lymphocyte. (A) Purified recombinant Der-p2 proteins as indicated were determined by Western blotting. Left panel, purified recombinant protein Der-p2 0.25, 0.5 and 1 µg. followed by staining with Coomassie Blue. Right panel, *D. pteronyssinus* antigen was recognized by C1 and C4 monoclonal antibody. (B) [^3^H]thymidine incorporation. B cells were pre-incubated with 1 mg/ml polymyxin B (PMB), an LPS inhibitor, and then treated with Der-p2 (1–10 µg/ml), LPS (1 µg/ml) or PMA (10 nM) for 72 hours. (C) Macrophage J774 or RAW264.7 cells pretreated with PMB then further stimulated with Der-p2 or LPS as indicated. Der-p2 did not activate NO production. PMB prevents LPS-induced NO production but not Der-p2. In D-F, B cells from healthy donor (D), asthma patient (AS) and atopic dermatitis (AD) patients (E, F) were stimulated for 12 hours with a non-cytotoxic dose of Der-P2. The expressions of proinflammatory mediators IL-1β , CXCL10, IL-8 and TNFα mRNA levels were detected by RT-PCR. Results shown are representative of at least three independent experiments.

Purified recombinant Der-p2 isolated from mite culture by affinity chromatography purification were shown in Coomassie Blue staining (Fig. 1A-left panel) or recognized by anti-mite Group-2 monoclonal antibodies C1 and C4 (Fig. 1A-right panel). We further tested whether Der-p2 induces B cell proliferation, which reflects the B cell activation. Der-p2 (1–10 µg/ml) treatment of cells for 72 hours significantly increased the B cell proliferation, which was determined by [^3^H]-thymidine incorporation, in a dose-dependent manner (Fig. 1B). Both LPS (1 µg/ml) and PMA (10 nM), as the positive controls, increased the B cell proliferation (Fig. 1B). LPS inhibitor polymyxin B (PMB, 1 µg/ml) markedly inhibited the LPS-induced cell proliferation, but did not affect the Der-p2-induced response (Fig. 1B). In addition, LPS markedly activated NO production in macrophages (J774 and RAW264.7 cells), which could be reversed by PMB, but Der-p2 did not activate NO production in these macrophages (Fig. 1C).

Der-p2 markedly increased the mRNA expressions of proinflammatory cytokines IL-1β, CXCL10, IL-8, and TNF-α in human B cells isolated from healthy donors (Fig. 1D) and patients with AS or AD (Figs. 1E and 1F).

**Figure 2:**
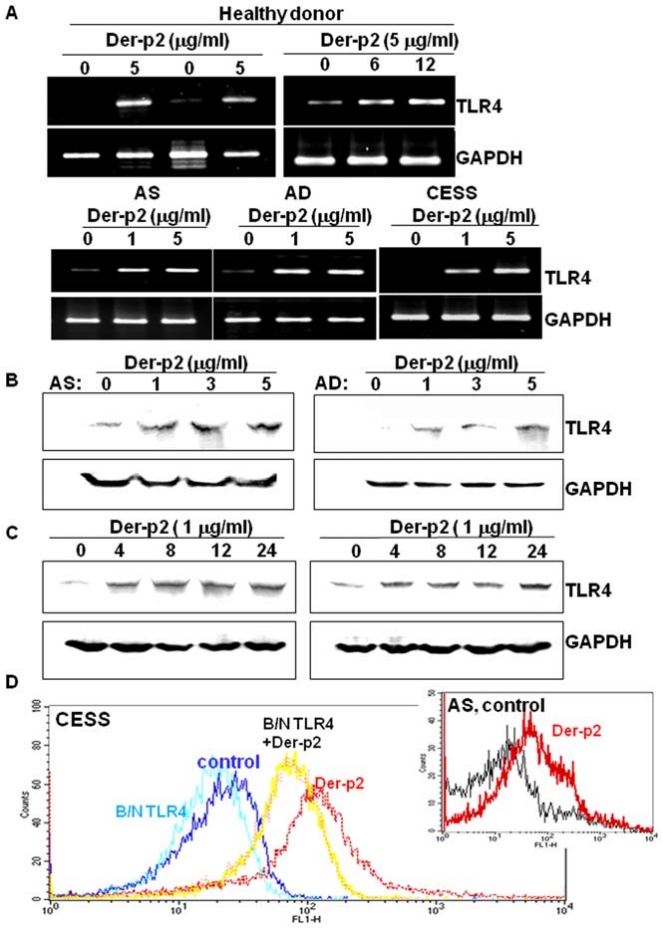
Mite allergen Der-p2 up-regulates TLR4 expression in human B cells. Human B cells from healthy donor, AS, AD or cell line CESS were treated with Der-p2 (1–5 µg/ml) for 4–24 hours. The TLR4 mRNA (A) and protein (B-C) expressions in B cells were determined by RT-PCR and Western blotting, respectively. (D) Cytofluorimetric analysis of TLR4 expression on B cells lines CESS treated with Der-p2. B cells were treated with Der-p2 (5 µg/ml) with or without blocking/neutralizing TLR4 antibody for 24 hours. Inside plot shows TLR4 expression in B cells from AS patient treated with Der-p2. Results shown are representative of four independent experiments.

Der-p2 (1–5 µg/ml) could also markedly increase the TLR4 mRNA (Fig. 2A) and protein (Figs. 2B and 2C) expressions in B cells in a dose- and time-dependent manner. Der-p2-blocking/neutralizing antibody was capable of inhibiting the induction of proinflammatory cytokines in Der-p2-treated B cells (data not shown). Moreover, the cytofluorimetric analysis showed that TLR4 was still expressed in B cells treated with Der-p2 in the presence of TLR4-blocking/neutralizing antibody (Fig. 2D).

### Der-p2 induces phosphorylation of ERK and dephosphorylation of p38

**Figure 3:**
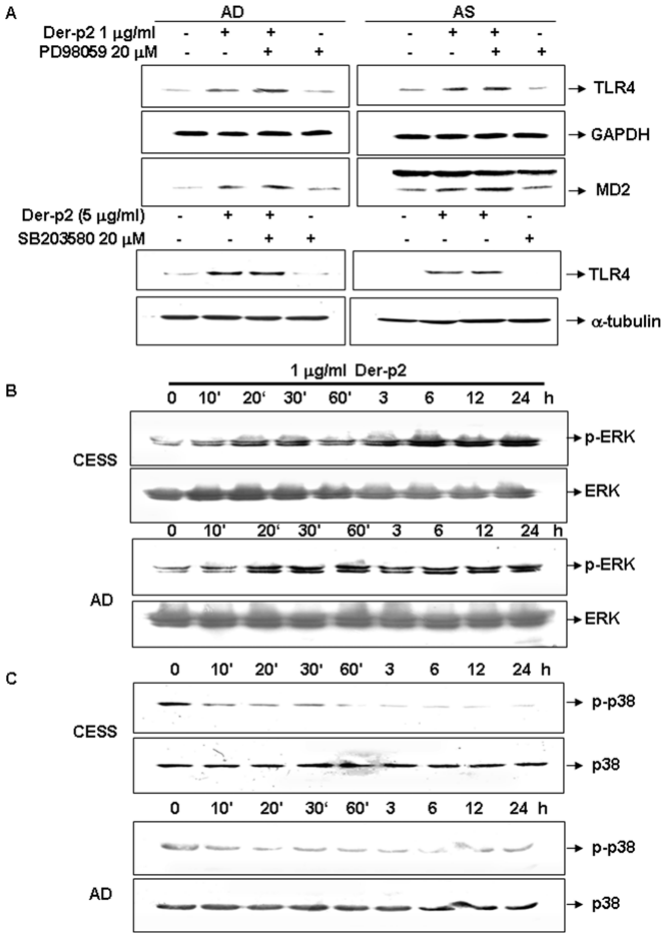
The changes of MAPK ERK and p38 phosphorylation and TLR4 protein expression in Der-p2-treated B cells. In A, B cells from AD patient and cell line CESS were cultured in serum-free medium and treated with Der-p2 (1 µg/ml) at the indicated times. Cell lysates were blotted and immunostained with anti-human phospho-p38, p38, phospho-ERK, and ERK antibodies. In B, human B cells were treated with Der-p2 (1 µg/ml) for 24 hours in the presence or absence of ERK inhibitor PD98059 (20 µM) or p38 inhibitor SB203580 (20 µM). TLR4 and MD2 protein expressions were assessed by Western blotting. Results shown are representative of at least four independent experiments.

As shown in Figure 3A, PD98059, a specific MAP kinase (MEK) inhibitor, effectively enhanced the increased protein expressions of TLR4 and its co-receptor MD2 in Der-p2-treated B cells. However, SB203582, a specific p38 inhibitor, did not affect the protein expressions of TLR4 and MD2 in Der-p2-treated B cells. Der-p2 triggered the phosphorylation of ERK in human B cells in a time-dependent manner (Fig. 3B). However, Der-p2 promptly induced p38 dephosphorylation in B cells (Fig. 3C). Der-p2 did not affect the activation of JNK (data not shown). These results indicate that ERK signaling may exert a negative regulatory effect on TLR4 expression in Der-p2-treated B cells.

### Der-p2 induces MKP-1-regulated p38 dephosphorylation and TLR4 expression

**Figure 4:**
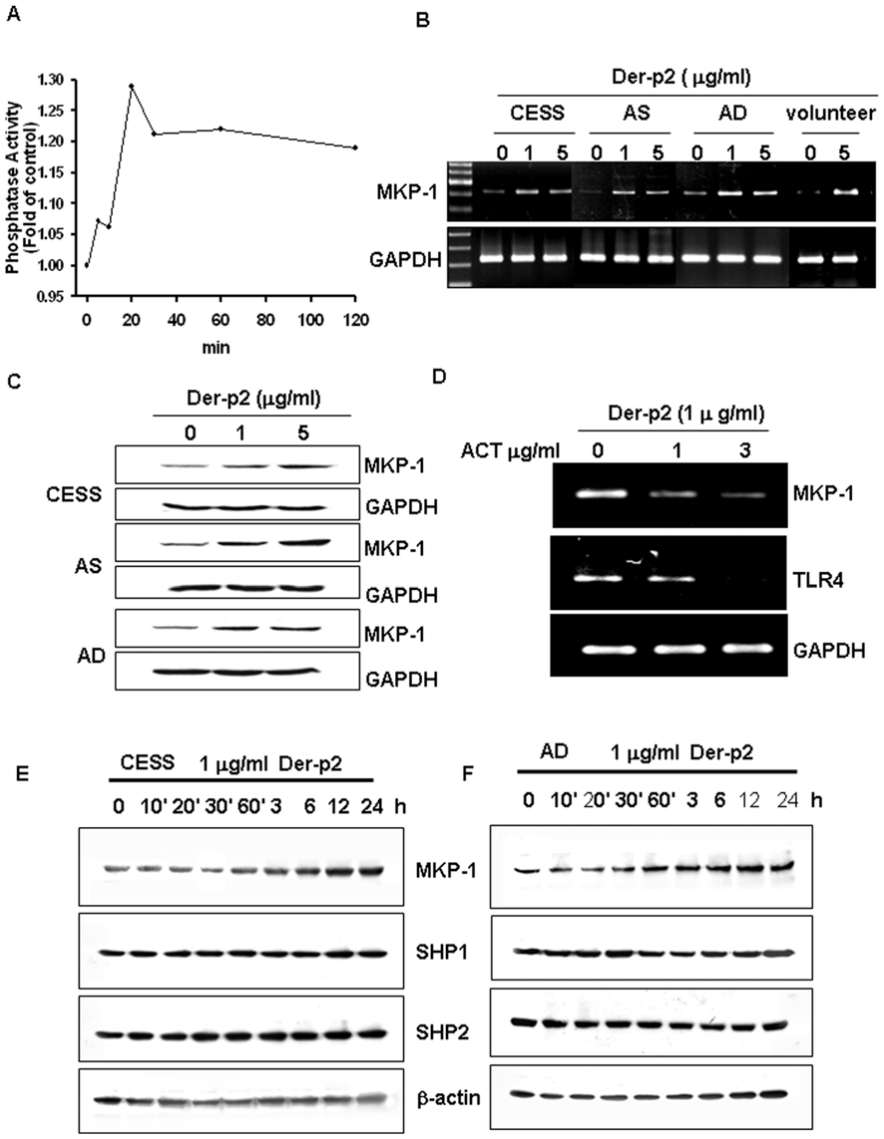
Der-p2 activates MKP-1 phosphatase activity and gene expression in human B cells. In A, B cells (CESS) were treated with Der-p2 (1 µg/ml) for various time intervals, and then the phosphatase activity of MKP-1 was detected as described in Materials and Methods. In B and C, B cells from healthy donor, AS patient, AD patient, or cell line CESS were stimulated with Der-p2 (1 and 5 µg/ml) for 3 h and 24 h, and then the MKP-1 mRNA (B) and protein (C) expressions were detected by RT-PCR and Western blotting, respectively. In D, B cells were treated with Der-p2 for 3 hours in the presence or absence of actinomycin D (ACT), and then MKP-1 and TLR4 mRNA expressions were assessed by RT-PCR. In E and F, B cells (CESS or primary cells from AD patient) were treated with Der-p2 (1 µg/ml) for 10 min to 24 hours, and then the MKP-1, SHP1, and SHP2 protein expressions were detected by Western blotting. Results shown are representative of at least three independent experiments.

MAP kinase phosphatase (MKP-1) is known to play a pivotal role in the deactivation of p38 and JNK. We found that Der-p2 effectively enhanced the phosphatase activity of MKP-1 (Fig. 4A) and the expressions of MKP-1 mRNA (Fig. 4B) and protein (Fig. 4C) in B cells. The Der-p2-enhanced MKP-1 and TLR4 mRNA expressions could be effectively reversed by actinomycin D (Fig. 4D). However, Der-p2 did not affect the protein expressions of phosphatases SHP-1 and SHP-2 in B cells. Moreover, we also tested the effect of Der-p2 on other SHP-1 and SHP-2 expressions in B cells (Fig. 4E and 4F).

**Figure 5:**
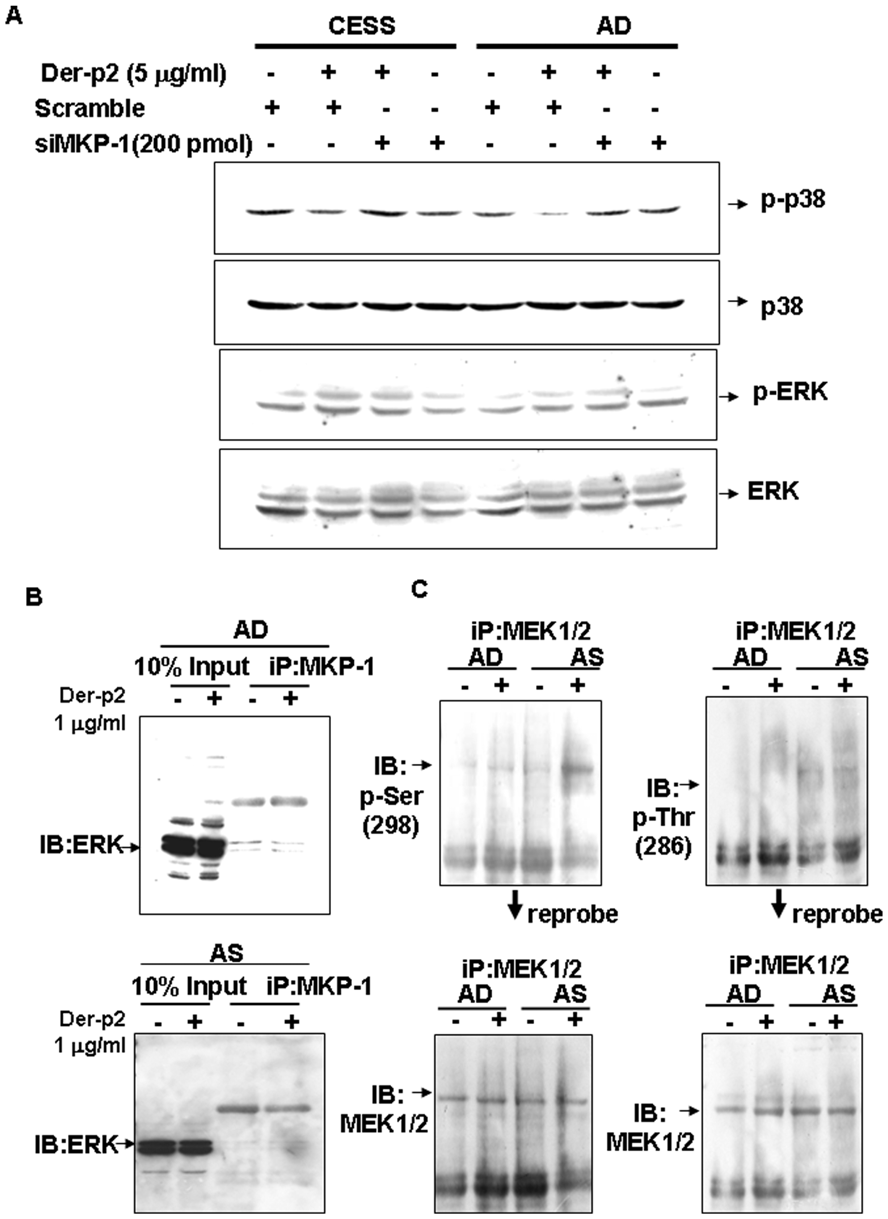
MKP-1 specifically regulates Der-p2-induced dephosphorylation of p38 but not phosphorylation of ERK in human B cells. In A, B cells from cell line CESS or AD patient were treated with Der-p2 (5 µg/ml) for 24 h in the presence or absence of siRNA-MKP-1. The phosphorylated and total protein expressions of ERK and p38 MAPK were determined by Western blotting. In B, B cells from AD or AS patients were treated with Der-p2 for 24 h. The cellular immunoprecipitation was prepared using a MKP-1 antibody, and then immunoblotting with phospho-ERK antibody was determined. In C, B cells were cultured in serum-free medium and treated with Der-p2 for 60 minutes. The cellular immunoprecipitation was prepared using a MEK1/2 antibody, and then immunoblotting with serine or thronine phosphorylation antibodies were determined. The immunoblots were also reprobed for total MEK1/2 protein detection. Results shown are representative of at least three independent experiments.

**Figure 6:**
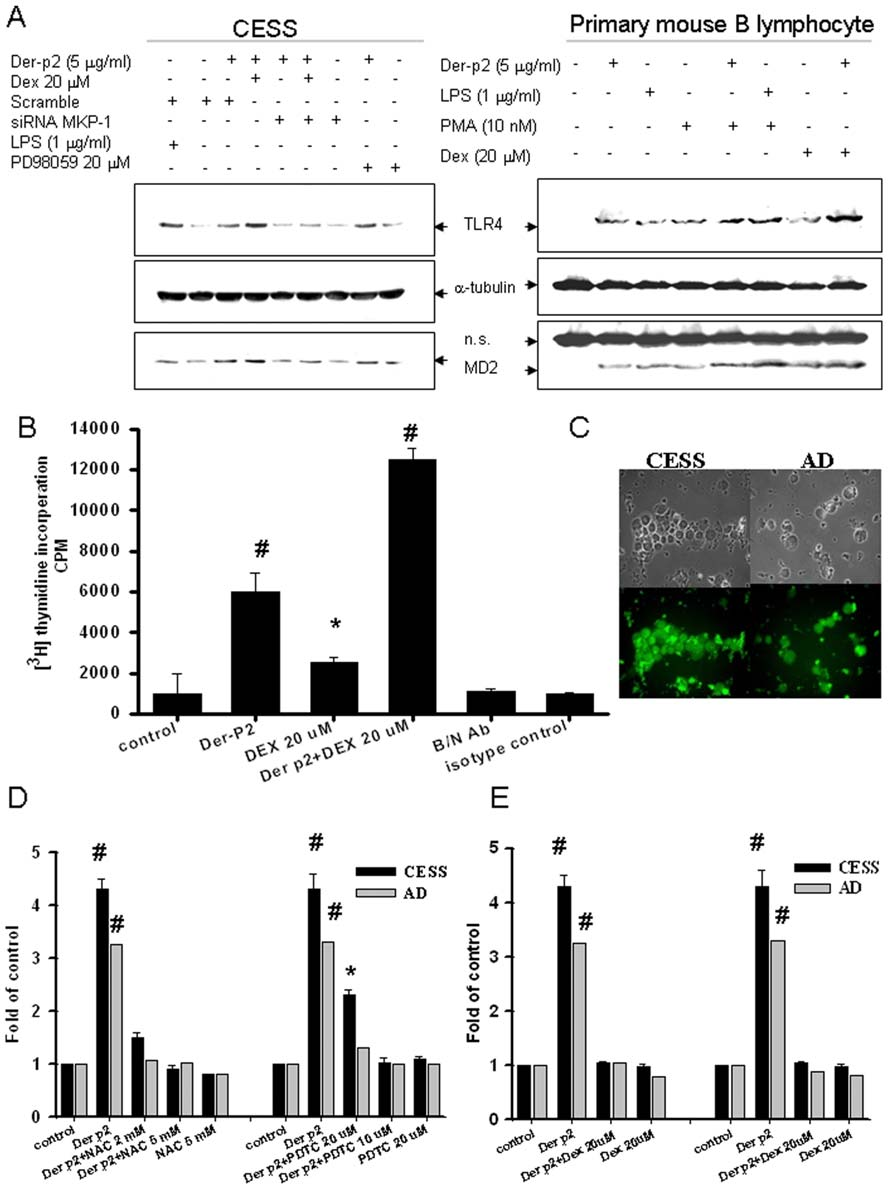
Dexmethasone augments Der-p2-induced MKP-1-regulated TLR4/MD2 expressions and B cell proliferation. In A, B cells (CESS) or primary B cells transfected with siRNA-MKP-1 or control siRNA (scrambled) were treated with Der-p2 for 24 h in the presence or absence of dexamethasone (Dex, 20 µM). TLR4/MD2 protein expressions were determined by Western blotting. Bacterial lipopolysaccharide or PMA were used as a positive control. B, B cells (CESS) were treated with Der-p2 (5 µg/ml) for 72 hours in the presence or absence of dexamethasone (Dex, 20 µM) or neutralizing Der-p2-antibody (B/N-Der-p2-Ab, 20 µg/ml), and then the cell proliferation was determined by [^3^H]-thymidine incorporation assay. Data are presented as mean±SEM (n = 5). In C, the efficiency of transfection in B cells was nearly 90%. In D, Der-p2 activates NFκB activity in human B cells. Human B cells from AD patient or CESS were transiently transfected with the NFκB-luciferase reporter plasmid constructs. NFκB activity was detected 24 hours after treatment with Der-p2 (5 µg/ml) in the presence or absence of N-acetylcysteine (NAC, 2 and 5 mM) or pyrrolidinedithiocarbamate (PDTC) (10 µM). In E, transfected B cells were treated with Der-p2 (5 µg/ml) for 24 hours in the presence or absence of dexamethasone (Dex, 20 µg/ml). Data are presented as mean±SEM (n = 5). # p<0.05 vs. control group.

We further found that Der-p2-triggered p38 dephosphorylation was abolished by MKP-1 siRNA, but Der-p2-induced ERK phosphorylation was not affected (Fig. 5A). The Der-p2-increased TLR4 and MD2 protein expressions were markedly reversed in siRNA-MKP-1-transfected B cells (Fig. 6A).

 The results of immunoprecipitation assay also showed that there was no crosstalk or interaction of MKP-1 with ERK during Der-p2 treatment (Fig. 5B). Moreover, activation of threonine or serine MEK1/2 provided dual-specificity kinase phosphorylation for the threonine and tyrosine residues on ERK. We found that the phosphorylation of MEK1/2 on threonine or serine residues by Der-p2 in B cells were not identical, which preferred the phosphorylation of serine residue, but not threonine residue (Fig. 5B).

 Glucocorticoids are known to induce the expression of MKP-1 in innate immune cells. As shown in Figure 6A, dexamethasone markedly enhanced the increased protein expressions of TLR4 and MD2 induced by Der-p2 (5 µg/ml) in human B cells. Transfection of siRNA-MKP-1 could effectively abolish the augmentation of TLR4 and MD2 protein expressions by dexamethasone plus Der-p2 (Fig. 6A). Dexamethasone (20 µM) also significantly enhanced the Der-p2-increased B cell proliferation, which could be effectively reversed by neutralizing Der-p2 antibody (Fig. 6B). The efficiency of transfection in B cells was nearly 90%, as shown in Fig. 6C. Moreover, co-stimulatory molecules in B cells are up-regulated during activation of B cells. The co-stimulatory molecules, such as CD 86, CD 40, and CD 69, were up-regulated in B cells by Der-p2 stimulation (supplementary data, Figure S1).

### Der-p2 triggers NF-κB activation in B cells

 As shown in Figure 6D, Der-p2 (5 µg/ml) triggered significantly greater NF-κB activation (3.2- to 4.3-fold) compared with the control in human B cells. Antioxidants N-acetylcysteine (NAC, 2 and 5 mM) or pyrrolidinedithiocarbamate (PDTC) (10 µM) effectively reversed the Der-p2-triggered NF-κB activation in B cells. Moreover, dexamethasone (20 µM) could significantly abrogate the Der-p2-triggered NF-κB activation in B cells (Fig. 6E). These results indicate that dexamethasone possesses the ability to inhibit Der-p2-induced NF-κB activation in B cells.

## Discussion

**Figure 7:**
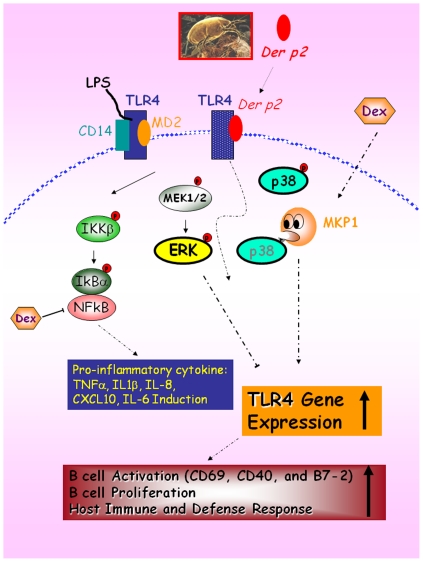
Schematic diagram of the role of TLR4 in the context of Der-p2-induced B cells activation. The signaling cascade involved in simultaneous activation of MEK1/2-ERK and dephosphorylation of p38 signaling in human B cells. As indicated, Der-p2 specific up-regulation of MKP-1 results in dephosphorylation and inactivation of p38 MAPK. However, Der-p2 activated phosphorylation of ERK simultaneously. The ERK inhibitor, PD98059 augments TLR4 expression, and was found to be a negative regulator of TLR4. Moreover, B cells evoke the Der-p2-induced expression of the key cytokines, including IL-1β, CXCL10, IL-8 and TNF-α, thus setting in motion an adaptive immune response and thereby contributing significantly to host immune and defense responses.

The results of the present study revealed a novel and important finding which sheds light on the involvement of a signaling cascade in the simultaneous activation of MEK1/2-ERK and dephosphorylation of p38 signaling in human B cells. Der-p2-specific up-regulation of MKP-1, in turn, results in dephosphorylation and inactivation of p38 MAPK. However, Der-p2 activated phosphorylation of ERK simultaneously. The ERK inhibitor, PD98059 augments TLR4 expression, and is a negative regulators of TLR4. Moreover, B cells evoke the Der-p2-induced expression of the key cytokines, including IL-1β, CXCL10, IL-8 and TNF-α, thus set in motion an adaptive immune response thereby contributing significantly to host immune and defense responses. (Fig. 7)

B cells are generated throughout life by differentiation from hematopoietic stem progenitors and constitute approximately 15% of peripheral blood leukocytes [25]–[27]. It has been shown that human HSP60 induces native mouse B cells to proliferate and to secrete IL-10 and IL-6 via the TLR4 and MyD88 signaling pathway [28]. Hammad and colleagues found that the absence of TLR4 in structural cells abolished house dust mite-driven allergic airway inflammation [29]. A recent study by Trompette and colleagues suggested that the propensity of Der-p2 to be a target of adaptive immune responses is due to the fact that Der-p2 promotes TLR4 signalling and thus displays auto-adjuvant properties [30]. Liu and colleagues have also shown that Der-p2 induces nitric oxide production in alveolar macrophage cell lines via CD14/TLR4 [31]. In addition, a previous study by Poltorak and colleagues showed that the mammalian TLR4 protein is adapted primarily to subserve the recognition of LPS and presumably transduces the LPS signal across the plasma membrane [32]. Hoshino and colleagues have also demonstrated that macrophages and B cells from TLR4-deficient mice do not respond to LPS, indicating that TLR4 is the gene product that regulates LPS response [33]. A recent study also showed that a lack of TLR4 is associated with an even stronger inflammatory response in the lung, and this suggests that TLR4 is a negative regulator in noninfectious lung inflammation [34]. Gruber and colleagues demonstrated that Der-p2 is composed of β-sheet and random coil and exhibits crystal structural homology with MD-2 [35]. MD-2, which is physically associated with the extracellular part of TLR4, is necessary for the intracellular signaling cascade after LPS binding. However, the role of B cells in enhancing host immune and defense response against invading mite allergens is still unclear. The molecular basis for B cell activation and TLR4/MD-2 induction in human B cells under Der-p2 exposure also not completely understood. The present work found that the B cell activation and proliferation and TLR4/MD2 expressions in human B cells could effectively be induced by both Der-p2 and LPS. Both TLR4 and MD-2 appear to be essential for B cell responses to Der-p2, at least with respect to the induction of immune mediators that are associated with chronic inflammation or recurrent infection disease. Moreover, proinflammatory cytokines, such as IL-1, IL-8, CXCL10, IL-6, and TNF-α, are known to be released within hours after inflammation challenge and serve as unspecific alarm signals, as they are released during viral infection, asthma and atopic dermatitis. Here, we found that Der-p2 could also markedly induce the expressions of proinflammatory cytokines in human B cells. Taken together, these findings indicate that Der-p2 is capable of triggering B cell activation and TLR4 induction in human B cells. The induced TLR4 may initiate the host immune and defense responses against Der-p2 infection.

MAP kinases are critical mediators of innate immune responses. MKP-1, a threonine/tyrosine dual-specificity phosphatase, efficiently suppresses MAP kinases p38 and JNK phosphorylation in murine macrophages and other cell types [36]–[40]. MKP-1 has been demonstrated to be a pivotal feedback control regulator of the innate immune responses and plays a critical role in suppressing endotoxin shock [41]. Bhattacharyya and colleagues have shown that dexamethasone inhibits LPS-induced TLR4-mediated inflammatory responses in macrophages through the glucocorticoid receptor-related MKP-1-induced p38 inhibition [42]. Wang and colleagues have further found that dexamethasone induces the expression of MKP-1 *in vivo*, and protected mice from mortality caused by a relatively high dose of LPS [43]. Dexamethasone has also been shown to profoundly induce MKP-1 expression in epithelial cells which results in repression of the p38 MAPK pathway, leading in turn to repression of the TNFα-induced NF-κB-dependent transcription [44]. In the present study, we demonstrate that Der-p2 induces phosphorylation of ERK1/2, as well as dephosphorylation of p38/MAPK in human B cells under Der-p2 exposure. Der-p2 did not affect the expression of JNK in B cells. ERK inhibitor PD98059 effectively enhanced Der-p2-induced TLR4 expression in B cells, indicating that ERK may be a negative regulator of TLR4 induction. The upstream regulator of ERK, MEK1/2, can activate p44 and p42 ERK/MAP kinase by phosphorylation of both threonine and tyrosine residues at sites located within the activation loop of kinase subdomain VIII. We further found that Der-p2 could activate the phosphorylation of MEK1/2 at serine but not at threonine residue site in human B cells. Moreover, the present work also found that knockdown MKP-1 by siRNA effectively reversed p38 dephosphorylation and TLR4/MD2 expressions induced by Der-p2 in B cells. These finding demonstrate that MKP-1 is involved in the dephosphorylation of p38 and TLR4/MD2 expressions induced by Der-p2 in B cells. Moreover, dexamethasone-enhanced TLR4/MD2 induction by Der-p2 could also be abrogated by siRNA MKP-1, suggesting a novel cellular and molecular mechanism for dexamethasone in the regulation of innate immune responses.

In conclusion, this study demonstrates for the first time that Der-p2 is capable of triggering human B cell activation and TLR4 induction. Der-p2 markedly induced the expressions of several key cytokines including IL-1β, CXCL10, IL-8, and TNF-αin B cells. Der-p2 could also enhance NFκB activity, indicating that Der-p2 may exert its influence through NFκB activation to induce the production of proinflammatory cytokines. Moreover, Der-p2 specifically up-regulated MKP-1 expression and activity in human B cells, which in turn, resulted in p38/MAPK dephosphorylation, triggering TLR4 induction.

## Materials and Methods

### Cells and Culture

 Human B cell line CESS cells were a gift from Dr. KI Lin (Academia Sinica, Taiwan). Cells were maintained in RPMI-1640 medium containing 10% heat inactivated FCS and streptomycin/penicillin in a humidified 5% CO_2_ atmosphere. In some experiments, the human B cells were isolated from the blood of healthy persons provided by the Taiwan Blood Services Foundation and from patients with asthma or atopic dermatitis after obtaining written informed consent. The study was approved by the Research Ethics Committee of Taichung Veterans General Hospital.

### Ethics statement

 State the consent and approved documents was obtained. All animal experiments were performed according to the National Institutes of Health Guide for Care and Use of Experimental Animals and approved by National Chung Hsing University Animal Care and Use Committee (Animal welfare assurance No. NCHU-98-102). In addition, all human sample subjects provided informed consent, the protocols, and all research involving human participants were approved by the Institutional Review Board of Taichung Veterans General Hospital. State the ethics committee specifically approved document (TCVGH-C07261-3). Measures were taken to clear from the approved documents were obtained that had accumulated over the past two years.

### Purification of naive B cells

Human PBMCs were prepared by density centrifugation (Ficoll-Paque). In some experiments, mouse spleen cell suspensions were depleted of RBC by treatment with red blood lysis buffer (Sigma-Aldrich). B cells were then purified by negative selection with a B cell isolation kit containing biotin-conjugated monoclonal antibodies to CD43, CD4, and Ter-119 (Miltenyi Biotec). This procedure routinely yielded B cell preparations that were >95% positive for the B220 marker as determined by FACS analysis.

### Der-p2 preparation

Purified recombinant protein Der-p2 was prepared as previously described [6]. The endotoxin contamination was tested by Pyrochrome Limulus Amebocyte Lysate assay (Associates of Cape Cod). The endotoxin level was low (0.0074±0.01 ng/mg).

### Proliferation assay

B cells were cultured with or without Der-p2 or LPS (*Escherichia coli* strain 055:B5; Sigma-Aldrich) treatments. Cells were pulsed with 1 µCi of [^3^H]thymidine and incubated for a further 24–72 h, and then were harvested. The incorporation of [^3^H]thymidine was measured by scintillation counting.

### Nitric oxide (NO) production

NO production was estimated by measuring nitrite accumulation in culture medium using the Griess method.

### Flow cytometric analysis

Both surface activation and costimulatory molecules were detected by flow cytometry. B cells (2×10^5^/well) were cultured with FITC-conjugated anti-mouse CD86, CD80, or CD69 or PE-conjugated CD40, TLR4 (BD PharMingen) for 30 min on ice. After washing, stained cells were quantified using the FCScan system and CellQuest software (BD Biosciences).

### Reverse transcription-polymerase chain reaction (RT-PCR)

Cells were homogenized with 1 ml of TRIzol reagent (Invitrogen). The total RNA was isolated according to the manufacturer's protocol. The first standard cDNA was synthesized by the extension of (dT) primers with 200 units of SuperScript II reverse transcriptase (Invitrogen) in a mixture containing 1 µg of total RNA digested by RNase-free DNase (2 units/µg of RNA) for 15 min at 37°C. Then the cDNA served as a template in a PCR using the PerkinElmer DNA Thermal Cycler (model 480). The sense primers (5′ or 3′) were: MKP-1, 5′-GCTGTGCAGCAAACAGTCGA-3′, IL1-β 5′-AAACAGATGAAGTGCTCCTTCCAGG; TNFα, 5′-GGCTCCACCCTCTCTCCCCTG; TLR4, 5′-CTTATAAGT GTCTGAACTCCC; IL-8, 5′-TTGGCAGCCTTCCTGATTTCT-3′; β-actin 5-GATGATGATATCGCCGCGCT; CXCL10, 5′-GCTTAGACATATTCTGAGCCTAC-3′, at a concentration of 0.4 µM. The amplification cycles were as follows: 94°C for 60 s, 55°C for 60 s, and 72°C for 90 s. Then the PCR products were subjected to electrophoresis on a 2% agarose gel after 30 cycles. The electrophoresis products were visualized by ethidium bromide staining. The mRNA of β-actin was used to control sample integrity and loading.

### Western blotting

Whole cell lysates were prepared as described previously [45]. After blocking, the blots were incubated with antibodies for anti-human TLR4, MD2, p-ERK, ERK, p-p38, p38, MKP-1, and β-actin (Santa Cruz Biotechnology) in PBS with 0.1% Tween 20 for 1 h followed by three 10-min washes in PBS with 0.1% Tween 20. The membranes were then incubated with horseradish peroxidase-conjugated secondary antibodies for 60 min. Detection was performed with ECL (Amersham), and chemiluminescence was measured by Kodak X-Omat film.

### MKP-1 phosphatase activity

MKP-1 activity was measured using immunoprecipitated MKP-1. Cells were lysed in lysis buffer, and 500 µg of supernatant protein was incubated with 2 µg of antiphosphorylated MKP-1 antibody or IgG at 4°C for 2 h, followed by incubation with protein A/G PLUS overnight. Immunoprecipitates were washed three times with cell lysis buffer. Phosphatase activities of immunoprecipitated MKP-1 were analyzed using *p*NPP protein phosphatase assay kit (AnaSpec).

### Transfection, luciferase reporter, and siRNA

Cells were transfected with plasmid using Lipofectin (Invitrogen Life Technologies). For promoter reporter assays, cells were cotransfected with either pNF-κB-Luc or pRL-TK (Renilla luciferase control). Firefly and Renilla luciferase activity was determined by Dual Luciferase Kit (Promega Corporation) following the manufacturer's protocol. A specific siRNA (MWG Biotech, Germany) was used to specifically knock-down the MKP-1 expression. After 48 h of transfection, cells or cell lysates were collected and analyzed for luciferase activity or protein expression, respectively.

### Statistical Analysis

The values are presented as mean±SEM. All analyses were performed by analysis of variance followed by Fisher's least significant difference test. *P* values less than 0.05 was considered statistically significant.

## Supporting Information

Figure S1 **Der-p2 up-regulated costimulatory molecule expression.** B cells from C57BL/6J mice were treated with Der-p2 (5 µg/ml). After 24 hours, the cells were stained with antibody FITC-conjugated anti-mouse CD86, CD80, or CD69 or PE-conjugated CD40, and detected by flow cytometry. The block line shows control unstimulated cells, and the red line shows Der-p2 (5 µg/ml)-treated cells. The pink line shows Der-p2 (5 µg/ml) combined with Dex 20 µM, and the green line shows Der-p2 (5 µg/ml) combined with PD98059 (20 µM). The results are representative traces of at least three experiments.
(TIF)
