# Supplementary figures and images for: Mite Allergen Der-p2 Triggers Human B Lymphocyte Activation and Toll-Like Receptor-4 Induction
Source: PLoS One. 2011 Sep 6;6(9):e23249. doi: 10.1371/journal.pone.0023249 (PMC3167811; doi:10.1371/journal.pone.0023249)

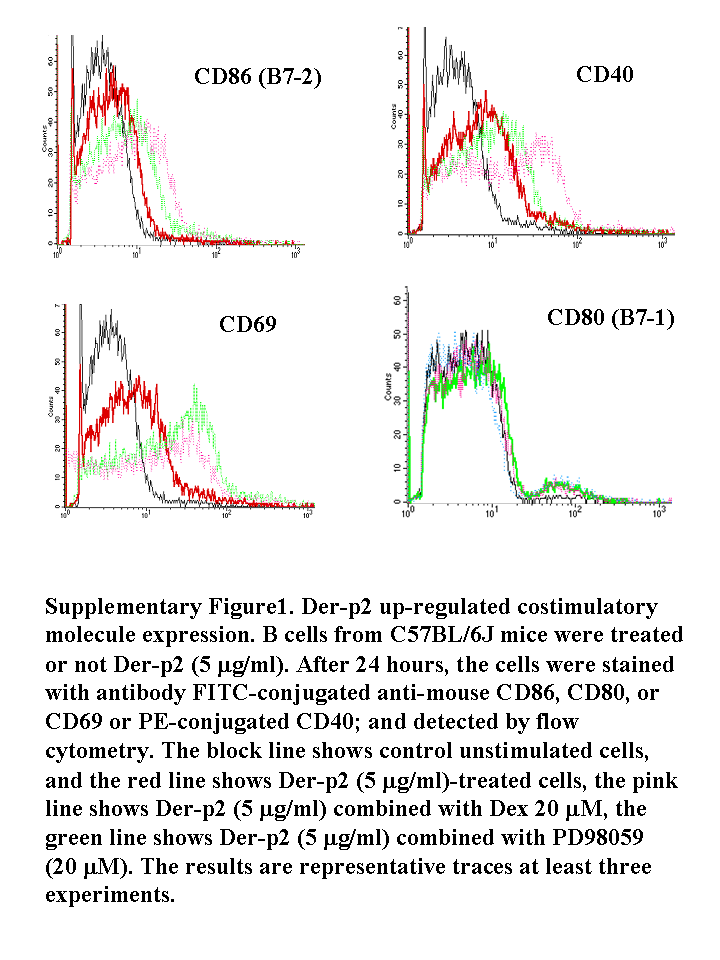

Supplement: Figure S1 — Der-p2 up-regulated costimulatory molecule expression. B cells from C57BL/6J mice were treated with Der-p2 (5 µg/ml). After 24 hours, the cells were stained with antibody FITC-conjugated anti-mouse CD86, CD80, or CD69 or PE-conjugated CD40, and detected by flow cytometry. The block line shows control unstimulated cells, and the red line shows Der-p2 (5 µg/ml)-treated cells. The pink line shows Der-p2 (5 µg/ml) combined with Dex 20 µM, and the green line shows Der-p2 (5 µg/ml) combined with PD98059 (20 µM). The results are representative traces of at least three experiments. (TIF) [file pone.0023249.s001.tif]
